# Paediatric Pain Medicine: Pain Differences, Recognition and Coping Acute Procedural Pain in Paediatric Emergency Room

**DOI:** 10.3390/medicina54060094

**Published:** 2018-11-27

**Authors:** Gabija Pancekauskaitė, Lina Jankauskaitė

**Affiliations:** 1 Faculty of Medicine, Medical Academy, Lithuanian University of Health Sciences, LT-44307 Kaunas, Lithuania; lin.jankauskaite@gmail.com; 2 Department of Pediatrics, Medical Academy, Lithuanian University of Health Sciences, LT-50161 Kaunas, Lithuania

**Keywords:** paediatric pain, acute pain, procedural pain, pain assessment, management, nonpharmacological

## Abstract

Paediatric pain and its assessment and management are challenging for medical professionals, especially in an urgent care environment. Patients in a paediatric emergency room (PER) often undergo painful procedures which are an additional source of distress, anxiety, and pain. Paediatric procedural pain is often underestimated and neglected because of various myths, beliefs, and difficulties in its evaluation and treatment. However, it is very different from other origins of pain as it can be preventable. It is known that neonates and children can feel pain and that it has long-term effects that last through childhood into adulthood. There are a variety of pain assessment tools for children and they should be chosen according to the patient’s age, developmental stage, communication skills, and medical condition. Psychological factors such as PER environment, preprocedural preparation, and parental involvement should also be considered. There are proven methods to reduce a patient’s pain and anxiety during different procedures in PER. Distraction techniques such as music, videogames, virtual reality, or simple talk about movies, friends, or hobbies as well as cutaneous stimulation, vibration, cooling sprays, or devices are effective to alleviate procedural pain and anxiety. A choice of distraction technique should be individualized, selecting children who could benefit from nonpharmacological pain treatment methods or tools. Nonpharmacological pain management may reduce dosage of pain medication or exclude pharmacological pain management. Most nonpharmacological treatment methods are cheap, easily accessible, and safe to use on every child, so it should always be a first choice when planning a patient’s care. The aim of this review is to provide a summary of paediatric pain features, along with their physiology, assessment, management, and to highlight the importance and efficacy of nonpharmacological pain management in an urgent paediatric care setting.

## 1. Introduction

The International Association for the Study of Pain defines pain as “an unpleasant sensoric and emotional experience linked to confirmed or possible tissue injury” [1]. Acute pain is one of the major complaints in paediatric emergency rooms (PERs) [2]. Conditions such as acute paediatric minor and major traumas, injuries, abdominalgia, acute headache, etc., are associated with a pain of different intensities. Paediatric pain itself is a challenge for a child, his or her parents, and medical staff in a PER. Procedures in PERs represent one of the most common sources of acute painful stimulus in a child. Studies have shown that up to 80% of emergency room (ER) patients undergo painful diagnostic procedures, such as venepuncture, intravenous insertion and removal, capillary sampling, shots, oral and nasal suctioning, tape removal, or urine sampling. Painful procedures are usually unexpected, so it intensifies hospital-related stress and anxiety leading to unpleasant experiences and bad memories associated with medical settings that can adversely affect procedure outcomes [3]. It may further influence future visits and intensify a patient’s fears. The simple thought of visiting hospital settings can additionally provoke distress for a child. Procedural pain leading to anxiety and fear can be prevented or largely reduced. However, many evidence-based interventions, tools, and methods remain severely underutilized in the paediatric population [4,5,6].

It must be understood that a child’s pain is very different from that which is experienced in adults. Different emotional and psychological factors can affect the child’s pain comprehension and stimulate his/her response. The first step to good procedural pain reduction is initial pain and distress evaluation. Various scales have been created to assist physicians in understanding pain in children of different ages. It is crucial for medical staff working in PERs to timely recognize signs and symptoms of pain during the procedures and determine if they are pain related, keeping in mind other possible factors as fear, distress, or manipulation [7]. Paediatric procedural pain is often underevaluated or not assessed at all, leading to inadequate pain management. Stevens et al. reported that in only 28% of paediatric pain cases was pain documented and did children receive pain management associated with a painful procedure [6]. Ali et al. revealed that 60% of urinary catheterizations and 53% of intravenous line placements were performed without any analgesia in Canadian paediatric emergency rooms [5]. According to a study performed by MacLean et al., <1% of patients undergoing venepuncture or intravenous line placement received topical anaesthesia; the rest of them had no pharmacological pain management documented [4].

Assessment of acute paediatric pain is extremely difficult. Along with the crowded ER environment and medical staff of different levels of training, variations in children’s age and gender, development and communication level, different personalities and temperaments, individual clinical condition, and his or her personal response to a painful stimulus should be considered. Moreover, previous experiences related to primary care and/or hospital settings must be thought about as well [7]. Medical staff education and skills on pain identification and management can vary from ER to ER and it can be another important factor in pain medicine. Inadequate knowledge or skills during training or continuing education, personal bias about (e.g., “pain medication will mask clinical condition”) and attitude towards pain, underuse of pain-scoring tools, or failure to recognize the need of medication as well as a lack of standards for pain reduction or local healthcare system can be alone or together obstacles to optimal pain control. Another effector to take in consideration is presence of patient’s parents, caregivers, or relatives. The vast majority of children are accompanied by carers who can contribute directly or indirectly to anxiety and pain. Thus, paediatric emergency settings must be child and family oriented. Parents values, personal beliefs, as well as misconceptions (e.g., “children do not feel as much pain as adults”) can result in inadequate pain management [8,9]. An additional barrier to optimal pain control can be increased concerns regarding pharmacological pain medication and application methods. This results in lower doses of painkillers at home, primary care, ER, and hospital settings. Moreover, it can prevent or limit medical staff from prescribing an adequate analgesia during painful procedures. Besides, parents can be affected by their children’s distress, or their stressful behaviour can increase distress and pain in their children [10,11].

Pain management is extremely important for newborns and infants. Insufficient pain relief may cause long-term changes in pain understanding and perception and determine specific pain-related behavioural expressions. Procedural-pain-associated stress and discomfort have long-term negative effects on patients and their parents/caregivers. It may contribute to eating and sleeping disorders, provoke post-traumatic stress disorder, diminish social skills, or increase fears [7]. The medical staff is responsible for well-timed and adequate pain management as well as stress and anxiety control and all-around patient safety. Lately, more and more data focus on nonpharmacological acute pain reduction methods as the first and one of the most important parts in managing paediatric pain, especially in an urgent care environment. When used correctly, nonpharmacological measures are able not only to control procedural-related pain and anxiety but also lower doses of required medication and in some cases even avoid pharmacological painkillers [12,13].

## 2. Neurobiology and Physiology of Pain—It Is a Different Feeling!

Nociceptive pathways in early life are not a lessened version of an adult ones. Neurobiological studies of child and infant pain have been neglected for years. To date, there has been an advancement in understanding of pain pathways, important mediators, and responses to noxious stimuli.

The starting point of the pain pathway is irritation of nociceptors. Nociceptors, as pain-sensitive axon terminals, are spread throughout most body tissues. They are responsive to thermal, chemical, and mechanical stimuli after birth [14]. Noxious stimuli cause tissue injury and activate nociceptors indirectly when chemical substances (ions of potassium, serotonin, bradykinin, histamine, prostaglandins, leukotrienes, or substance P) are released. These agents trigger axon terminals and turn mechanical or chemical stimuli into action potentials, which is the start of the pain pathway. The impulse is driven towards the central nervous system (CNS) by the axon of the first neuron. When the spinal cord (SC) is reached, the first neuron forms a synapse with a cell of the dorsal horn. The information is sent to the thalamus through the anterolateral system. The neurons of the SC dorsal horn form synapses with neurons of the ventral posterior nucleus and the impulse of pain is sent further to the primary and secondary somatosensory cerebral cortex. Together with emotional and cognitive components, nociceptive impulses form the full conception of pain in the cerebrum [15]. 

The pathway of pain in the body of a child has its own features which determine different sensations and perceptions of pain. The misconception and misunderstanding exist that our youngest patients do not feel pain, or that it is not as strong as in an adult. However, the nociceptive system starts functioning already at the 20th week of gestation [16]. To fully understand differences in children’s pathway of pain, it is convenient to compare the pathways of a premature newborn and an adult (Table 1). The pain pathway changes during growth and development. Both premature newborns and adults have fully developed nociceptors; however, junctions between nociceptive neurons and the ones in the spinal cord do not function the same. Terminals of the first neuron in the pain pathway form synapses with neurons of the SC in the body of an adult. The nociceptive neurons do not form any specific structure in the SC of preterm neonates, meaning that some axons cover each other in the lamina II of the dorsal horn in the SC, which interrupts differentiation of tactile and nociceptive stimuli. Nociceptive and tactile stimuli are fully differentiated in the cerebral cortex in adulthood. Cortical responses to touch and pain are significantly different in preterm and term infants. Full-term infants demonstrate localized somatosensory responses to painful stimuli. Meanwhile, nonspecific neuronal bursts have been observed during electroencephalography (EEG) in preterm infants [17,18]. These studies confirm that the premature newborn brain is more sensitive and can poorly distinguish noxious and innocuous stimulation [19]. Holsti et al. showed that diaper changes can provoke the same bio-behavioural responses as blood collection due to prior pain experiences in preterm infants [20], The noxious stimuli activate a neuroimmune response, making nociceptive reflexes of the SC and microglial reactions stronger in a newborn’s body. Nevertheless, reactions stop in a predominantly anti-inflammatory phase and neuroinflammation is not developed. The immune system undergoes significant maturation postnatally and it can be “primed” by various noxious stimuli during early life that lead to reactivation later on [21]. Data from an animal study performed by Beggs et al. showed that microglial reactions caused by pain in a neonatal period leave some irreversible changes even if it stops in a preinflammatory phase. These changes may still be present in adulthood, causing hyperalgesia and increased risk of developing chronic pain [22]. Nociceptive dorsal horn neurons are excited by various cytokines and growth factors [23]. Pain induces proinflammatory cytokines in the periphery and the CNS. Moreover, it overstimulates immature neurons, leading to alterations in the brain microstructure [17,18,24].

The impulse of pain is modulated by ascending and descending signals in a level of the SC. The descending pain modulatory system spreads from the nuclei of the cerebral trunk to the SC and consists of neurons which can produce serotonin and norepinephrine. Those neurons activate interneurons of dorsal horns that inhibit impulses of pain. However, the descending pain modulatory system is not active in the neonatal period because interneurons are not fully developed, and production of serotonin and norepinephrine is much slower compared to adults [25,26]. Another difference of the pain pathway in childhood is the different structure of neural fibers. Impulses of pain are transmitted through Aδ (myelinated) and C (unmyelinated) fibers. Even though myelinization is not complete in the nervous system (NS) of a child, it does not mean that they cannot feel pain or the perception of it is not as strong. Incomplete myelinization determines a slower signal rather than a weaker one. Still, a slower transmission of a pain impulse is fully compensated by a shorter length of pain pathway in the NS of a child [27]. 

Thus, there are some clear differences among nociceptive systems of children and adults. The number of nociceptors for one square meter of body surface is higher in a child’s body compared to an adult. The amount of neuromediators is higher as well, meaning a higher sensitivity to pain in childhood. The signal of pain is more intense and lasts longer for children when compared to adults [28]. Because of plasticity and specific features of children’s NS, prolonged or repeated pain at an early age increases the risk of neuron death or their disfunction in the future [22]. Many experimental and clinical studies note that painful stimuli have immediate and long-term consequences if untreated. Neonates exhibit a physiological increased sensitivity to pain. Acute pain in preterm newborns is associated with prolonged hyperalgesia leading to established or chronic pain [29]. Despite prematurity-associated clinical risk factors, procedural pain and stress in preterm infants is related to brain developmental disorders [24,30]. MRI studies have confirmed that greater exposure to procedural stress had primary and early effects on subcortical structures and secondary changes of white matter. Furthermore, studies using EEG or near-infrared spectroscopy (NIRS) registered cortical activity during the procedures in a neonatal intensive care unit (NICU) [17,18,19,31,32]. Rodent studies provide strong evidence that early exposure to painful stimuli is associated with structural and functional changes in the brain [33]. Few experimental studies have demonstrated that increased apoptosis in the neonatal rat brain resulted from acute pain during repeated injections [34,35,36]. Repetitive needle pricks significantly increased neuronal sensitivity in the contralateral dorsal horn [36]. In addition, it intensified postsurgical pain sensitivity in adult rats [37]. Early pain experience alters the descending pain control system from the brainstem. Besides, significant changes are observed postnatally in opioidergic receptor expression in the periaqueductal grey (PAG) [38]. Also, inflammatory pain at birth induced beta-endorphin and met/leu-enkephalin protein levels, leading to decreased opioid receptor expression in PAG in adulthood [39]. Chen et al. discovered that repetitive needlestick at an early age led to mechanical hypersensitivity and caused impaired spatial-memory retention in prepuberty rats. Both prepubertal and adult rats showed a decreased response to anxiety-induced stimuli. Moreover, hypothalamic–pituitary–adrenal axis function was dysregulated in young and adult rats that underwent frequent painful procedures as newborn pups [40]. Clinical studies performed by Grunau et al. identified that neonatal procedural pain and stress led to impaired brain development of preterm infants during the neonatal period and at school age [41,42,43]. Additionally, IQ at age 7 was altered in children who received painful procedures during the neonatal period [44]. At the age of 9–14 years, a greater activation of the somatosensory cortex was noted in children born preterm compared to those born full term [45]. Furthermore, psychopathologies in adulthood, such as depression or post-traumatic stress disorder, could be linked to repetitive untreated painful stimuli in the preterm period [46]. Thus, a timely used and properly chosen analgesic is highly important. However, clinical and experimental data on pharmacological pain treatment methods demonstrated that pain medication can have a neurotoxic effect in a child’s body [47]. Morphine is a well-studied and commonly used analgesic in NICUs or PERs. In experimental rodent studies analysing opioid exposure, impaired neuronal proliferation and survival was detected. Chronic exposure to morphine resulted in neuronal degeneration [48]. A clinical study by Steinhorn et al. revealed that low-dose morphine analgesia in NICUs induced early alterations in the cerebral structure [49]. Ferguson et al. concluded that children who received continuous morphine infusion in NICUs had a smaller head circumference and body size at the ages of 5–7 year. Moreover, these children performed poorly on short-term memory tests and were more prone to social problems [50]. Ketamine is another widely used anaesthetic, analgesic, and sedative agent. A number of studies have provided evidence for ketamine-induced neurotoxicity in the developing brain. An investigation by Jin et al. observed that ketamine resulted in greater and longer N-methyl-D-aspartate (NMDA) receptor channel blockade in immature neurons compared to mature ones [51,52]. Additionally, impaired proliferation of neuronal stem progenitor cells (NSPCs) has been described under the effect of ketamine. Moreover, NMDA triggered delayed neuroblast differentiation [53]. However, more clinical studies are needed to evaluate ketamine neurotoxicity mechanisms in children. Therefore, the pain itself and its evaluation and management are very important topics in paediatrics. However, they do not get enough attention, leading to poor knowledge and skills of medical staff and harmful actions for the patients in ER.

## 3. Acute Pain Assessment in Children

### 3.1. Pain Assessment Strategies

Good pain evaluation is an initial step contributing to pain prevention and/or early recognition leading to efficient pain management. There are three fundamental modes of pain assessment in the paediatric population: self-report, observational/behavioural, and physiological. Self-report is considered the gold standard [54]. However, its relevance directly correlates with a child’s age, development, and communication skills. In some cases, it can lead to subjective responses when the fact of manipulation of pain should be ruled out. An observational/behavioural approach could be used when pain and pain-related distress cannot be separated (e.g., cry or scream can be pain and/or fear dependent) [55]. Older children do often exhibit behaviours indicating pain (Table 2). However, their self-reports of pain do not always correlate with their behaviours [56]. Parent/caregiver observation and pain reports are of a high importance. Individuals differ in how they react and express pain. Thus, carers are essential to describe how a child normally behaves when in pain. Still, it cannot always be transferred to clinical utility, as it can reflect parents’/caregivers’ personality, individual perception, culture, and beliefs. Physiological parameters such as heart rate, blood pressure, respiratory rate, oxygen saturation, or salivary cortisol are indirect pain measurements [57]. These measurements cannot be used in isolation. Moreover, it is highly variable in the age group from 0 to 3 years [58]. Brudvik et al. analysed the differences between patient, parent, and doctor pain assessment in children aged 3–15 years. Pain intensity was self-reported by children. Parents and doctors assessed the child’s pain using an independent numeric rating scale (NRS). NRS is a verbal scale, for which a person is asked to rate his/her pain from 0 to 10, with 0 equal to no pain and 10 equal to worst pain possible. The results showed that doctors significantly underestimated the pain of paediatric patients compared to parents and patients themselves. Doctors were likely to assume that the patient’s reactions to pain were not in concordance with their medical condition. Also, the study determined that the doctors’ pain assessment improved with increasing levels of pain. However, only 42% of children with severe pain (NRS ≥ 7) received pain medication [59].

### 3.2. Pain Assessment Tools

There are many different pain assessment tools that have been developed. However, there is no single generally recommended tool or scale to evaluate children’s pain in an ER or outpatient setting. Besides, not all of them are used properly or timely. Pain evaluation has been factor oriented as follows: child-related, user-related, and structural. Children understand and express pain differently depending on their age stage (Table 3). Thus, the pain assessment must be age dependent [61]. The method of pain measurement is setting oriented and highly depends on medical staff experience as well as the individual patient [54]. Here, we provide a guide to the age-dependent pain evaluation tools which are most commonly used in our ER settings. We do not discuss scales for children with specific needs, such as cognitive impairment, etc.

**Figure 1:**
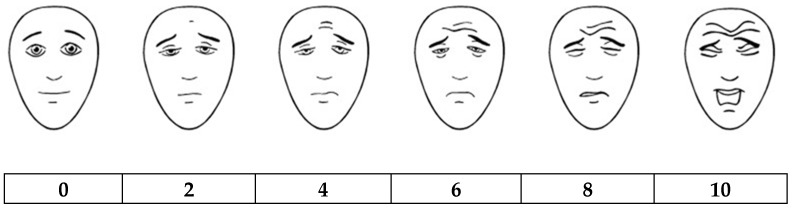
Revised Faces Pain Scale [65,66].

**Figure 2:**
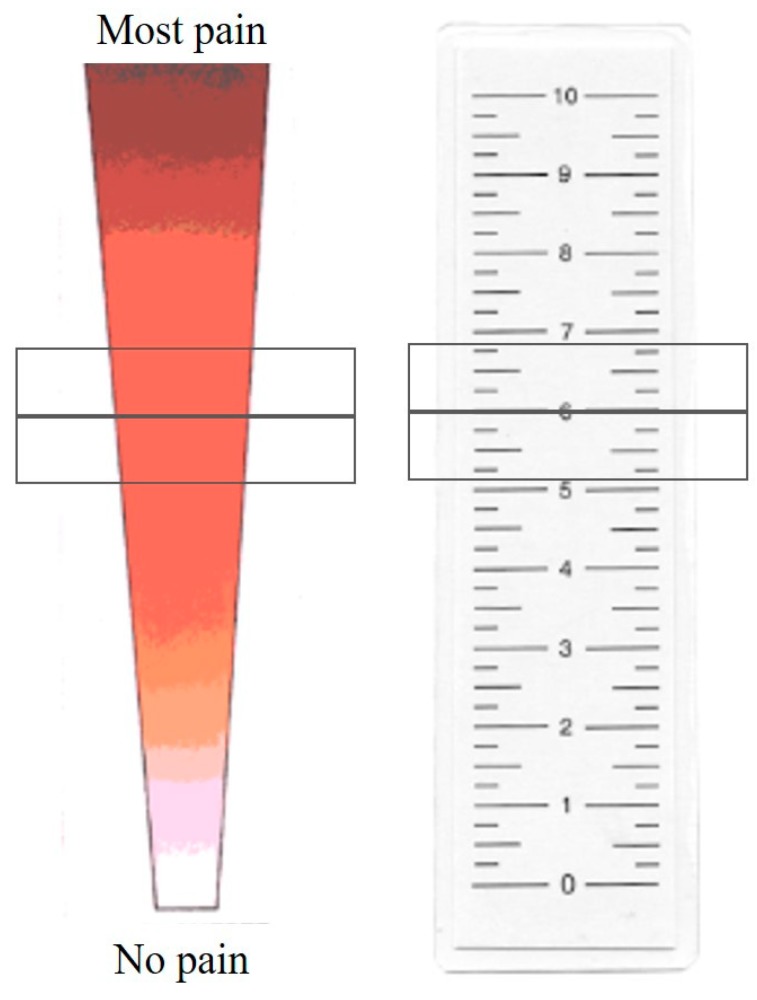
Color Analog Scale. Adapted from: McGrath, P.A. et al. A New Analogue Scale for Assessing Children’s Pain: An Initial Validation Study. *Pain* **1996** [70].

The Neonatal Infant Pain Scale (NIPS) was developed and validated in 1993 by Lawrence et al. [62]. Patients younger than 1 year of age are recommended to be assessed using this scale. Facial expressions, crying, breathing, position of arms and legs, and alertness of a baby are evaluated by a medical professional. The maximum score is 7. If the patient scores above 3, a nurse or a doctor should interpret it as that a newborn or a baby is in pain (Table 4). Another scale, called FLACC (a scale including Face, Legs, Activity, Cry, and Consolability evaluation (Table 5)) was developed by Merkel et al. in 1997 [63]. It is recommended for assessing pain of children from 2 months to 7 years old. Its reliability and validity were later confirmed by several studies [56,64]. Evaluation should be done in at least 5 min if a patient is sleeping and 1–5 min if a patient is active. A score from 1 to 3 demonstrates mild discomfort, 4–6 indicates average pain, and 7 and higher represents severe discomfort and/or severe pain; the maximum score is 10. The Revised Faces Pain Scale (FPS-R) developed in 2001 by Hicks et al. may be used for evaluating pain in children from 4 to 16 years of age. A scale of faces (Figure 1) is shown to a patient. It is explained that each face shows intensity of pain, with the most accurate face indicating pain-free condition and illustrating emerging and increasing pain. A child is asked to show the face representing his/her pain the best. Every face represents a certain score (0, 2, 4, 6, 8, 10) [65,66]. The Visual Analogue Scale (VAS) was developed in 1983 by Price et al. [67]. It is an easy and popular method that does not require any additional tools or long observation of a patient. The accuracy of VAS is close to the FLACC score. However, it can be used only for older children, starting from the age of 7 or 8. Besides, FLACC is superior for younger patients who have difficulties understanding the principles of VAS [68]. In VAS, a medical professional draws a line of 10 cm. One side of a line means absence of pain, the opposite side means unbearable pain. The patient must show the point on the scale representing his/her pain the best. The score of VAS is measured by millimeters or centimeters [67]. Yet, there are no clear cut-off points for children representing mild, moderate, or severe pain. So, sometimes it is hard to measure pain intensity and determine what treatment should be given. One suggested interpretation of VAS is to evaluate a 0–4-mm score as no pain, a 5–44-mm score as mild pain, a 45–74-mm score as moderate pain, and a 75–100-mm score as severe pain [69]. The Color Analog Scale (CAS) (Figure 2) was developed in 1996 by McGrath et al. The principle of CAS is close to VAS; the difference is that the 10-cm line is depicted in a colour transition. It is explained to the child which colour means the highest and lowest pain. Colours, rather than a plain line, may help a child to imagine a scale better so the answer can be more easily formulated. The score is measured by millimeters or centimeters as well [70]. A study performed by Le May et al. in 2018 compared FPS-R, CAS, and VAS. They included children aged 6–17 years with skeletomuscular trauma. The authors determined that all three pain scales have a strong correlation with each other, especially VAS and CAS. FPS-R, CAS, and VAS are all reliable enough to be used in PERs. However, CAS demonstrated a slightly higher responsiveness and reliability, so the authors recommended CAS for children 6–17 years old [71]. However, no other studies confirming these findings were found. Fernando et al. revealed that FPS-R was easier to understand than VAS in a sample of children 4–12 years old [72]. Moreover, medical staff reported that FPS-R helps them to assess children’s pain better than VAS. A study performed by Goodenough et al. demonstrated that children 4–16 years old preferred animated scales such as FPS-R and CAS as they were easier to use [73].

Nevertheless, it is essential to link scores to pain dynamics after treatment; a single score without a trend in response to treatment is of low value.

## 4. Acute Procedural Pain and Anxiety: A Complex Issue

Treatment of children during painful procedures differs from pain related to disease or other aetiology. Most of the time, it can be prevented and controlled by the medical staff or involved carers.

### 4.1. Preprocedural Preparation

One of the most important parts is preparation of a patient or his/her carers. It is known that underprediction of pain worsens subsequent procedural pain compared to overprediction [74]. The information about procedure should be age and development appropriate. Kolk et al. compared children with or without preparation for venepuncture. Patients and parents received sensory and clear procedural information. Additionally, children’s skin was numbed with local anaesthetics before the painful procedure. Results demonstrated that preparation was beneficial, showing significantly less distress during the venepuncture [75]. Suls et al. performed a meta-analysis and confirmed the dual process preparation hypothesis, meaning that a patient should be introduced to both the sensory and procedural expectations. A medical staff should explain what they will be doing as well as what the patient may feel (e.g., “You may feel a cold and wet pad while I clean your hand with antiseptic.”) [76]. To be clearer, procedures can be simulated on a doll, teddy bear, or mannequin. Children/adolescents or carers should be encouraged to ask questions and clarify the information at any stage of the procedure. Smith et al. interviewed 7–11-year-old children and noted that they can already identify their own information needs, so they should be allowed to ask questions themselves. Interviewed children expressed a wide variety of questions and demonstrated various levels of knowledge, from very limited to substantially high [77]. Children can be given choices but not control over the procedure. He or she and the parents or caregivers could be trained to cope with their feelings and promote positive emotions and behaviours. A different aspect is dealing with adolescents. They can tend to minimize or totally deny pain. Privacy is very important. Adolescents must be given an opportunity to have parents involved or not. They should be given freedom to control their feelings and stress under painful circumstances [78].

### 4.2. Role of a Parent

The majority of parents/caregivers express that they would choose to make their child’s procedure painless and stress-free and would agree to spend additional time in ER settings to reach a pain-free state [79]. ERs face time and resource constraints with high patient volumes. While carers are highly motivated to eliminate pain and distress their children face during procedures, they could be involved in processes such as preparation for a procedure and participate in pain and anxiety reduction and control. Most parents want to take part in a procedural process and comfort their child. Also, they should be recognised as a part of the healthcare team with clear instructions on their actions during the procedure. A study by Mangurten et al. demonstrated that the presence of parents does not interrupt medical care as reported by medical staff in an ER [80]. Parents noted that the ability to stay with a child during a medical procedure eased their fears. Moreover, they believed that parental presence helped their child. What is more, parents reported no traumatic memories three months later. It is essential to train adults to coach their children effectively during painful stimulus. A study by Blount et al. analysed children with or without training for breathing techniques for a painful procedure as well as parents who were coached on guiding their child through a breathing technique versus parents with no coaching [81]. As expected, the results showed that trained parents were more likely to engage in the child’s distraction during a procedure of immunization rather than untrained parents. In addition, trained children engaged in a breathing technique better and levels of distress were significantly lower in this group. Parents must be reminded that criticism and intimidation can result in higher procedure-associated distress levels in children [82,83]. Moreover, they must avoid unconscious projection onto their child associated with their own pain experiences. Children mirror their parents or carers in showing their own regulation of feelings, emotions, or physiological responses [84]. Frank et al. showed that 53% of the changes in a child’s distress during immunization was associated with parental behaviour. Jokes told by parents, commands on how to cope with stress, and nonprocedural talk increased the child’s ability to calm down. Furthermore, parents can be involved in special training programs or introduced to different booklets or books to learn more about childhood pain, preparation for painful procedures, hospital settings, and nonpharmacological coping techniques. In resource-limited settings, parental training was shown to be highly cost effective. In addition, the role of siblings is as important as that of the parents, especially before and after the procedure [85].

### 4.3. Resources: Environment

Child-friendly, calm, and comfortable surroundings are one of the major factors that may decrease distress and pain in children and their relatives. Specialised PERs can guarantee a safer and more peaceful environment for a child [7]. It is known that child-friendly medical settings may decrease anxiety in older children and calm their parents. One of the studies performed by Robinson et al. compared how caregivers and patients felt in a traditional ER environment versus an ambient lighting ER environment with interactive wall displays. The results showed that caregivers rated quality of care higher in the ambient ER group rather than the traditional one. Moreover, an ambient environment with interactive wall displays reduced children’s pain, anxiety, fear, and anger as reported by nursing staff [86]. Monti et al. evaluated parental stress before and after changing the environment of a paediatric ward (cartoons and other child-friendly images were painted on the walls) [87]. It resulted in a significant reduction of parental distress associated with their child’s hospitalization due to the colourful and child-friendly environment. Positioning of an infant or a child for their own comfort without restraint (sitting or hugging a parent) should always be a priority. Procedures should be performed in a comfortable place and a special procedural room [88].

### 4.4. Resources: Medical Staff

Special preparation of medical staff is crucial as well. Doctors, nurses, fellows, and play therapists should be included in a process in order to get the best of it. Previous experiences, personality, and analgesic preferences should also be taken into account while getting ready for the procedure. In any ER, with any procedure or using any kind of nonpharmacological technique or medication, staff must have enough knowledge and experience in pain recognition, preparation for the procedure, and the technique they will be using to reduce procedural pain and anxiety. Clinical effectiveness and possible deterioration should be monitored, and adverse events must be recognised and managed immediately. In ideal circumstances, all staff should know the simplest effective coping strategies regarding any age of a child and any family model. Staff should always offer support for parents and caregivers. It is highly recommended that emergency medicine departments should have a highly trained staff experienced in psychological techniques, for example, a play therapist. In case the nonpharmacological technique is used, medical staff should be able to determine if the application method is effective in managing the child’s current pain sensation. Pain needs must be assessed all through the procedure, as the last moments of it may determine how the child will remember the situation overall [89]. Moreover, all procedures should be preplanned; during and after the procedural period should be optimized regarding the psychological context. The language of the medical staff is another very important factor that may influence a patient’s experience. The content of the conversation must be well considered. Medical staff should avoid difficult medical phrases since it may scare and confuse children. Bribing or scaring children should not be used in a stressful medical environment, too. It may be useful for a short period of time; however, there will be no long-term benefits. Medical staff should stay honest with a child. Promises that cannot be kept or lies will ruin the trust and friendship that has been built between the staff and a child. As discussed before, adult coaching and distraction may decrease distress, but criticism, apologising, and saying “everything will be okay” may increase a child’s fear, stress, and pain [90]. Some of examples of language to use and avoid were listed in a supplement article by Cohen et al. (Table 6) [91].

### 4.5. Nonpharmacological Pain and Anticipatory Anxiety Treatment

Nonpharmacological pain and associated stress and anxiety management should always come first, and it must be offered to all children. It should be applied prior to or together with pharmacological treatment methods. First, it must be acknowledged that some pain medication is not recommended or safe for children. Midazolam and intranasal fentanyl are some of the most common medications used in PERs for pain control and anxiolysis. The most common indications are abscess incision or drainage, wound repair, and intravenous or Foley catheter insertion [92]. Even though these medications are proven to be safe for children when used in small doses, prolonged administration may cause long-term side effects. Duerden et al. determined that preterm infants exposed to midazolam repeatedly due to painful procedures had an altered growth and development of the hippocampus [93]. As previously discussed, different studies indicated early neurotoxic effects of analgesic medication leading to long-term neurodevelopmental changes.

Most nonpharmacological pain and anxiety treatment options have no age restrictions, are cheap, easily accessible, and can be used in any PER. Correct and timely application of nonpharmacological methods or tools may decrease the required dosage of painkillers, limit their side effects, as well as shorten the period of recovery [12,13]. Various methods and techniques exist and should be age and procedure oriented. Together with a calm, comfortable, and safe environment, parents and trained staff, different approaches can be used: distraction techniques and equipment such as deep breathing, guided imagery, music, iPad, or hypnosis; progressive muscle relaxation; cutaneous stimulation methods such as counter-irritation (e.g., cold, vibration, pressure); touch or thought stopping and suggestion; or sweet solutions. It is widely recognised that neurocognitive pathways directed towards pain perception can be disrupted if attention is drawn towards any kind of distraction task. Those tasks require the intentional use of cognitive resources when attention is voluntarily redirected to primary goals rather than pain [94,95,96,97].

Distraction techniques are divided into two groups: passive and active, which can be used separately or combined (Table 7). Passive techniques require the participation of medical staff or parents, whereas active techniques include the patient himself engaged during the procedure. Abdelmoniem et al. demonstrated that both active and passive distraction techniques separately or in combination were effective in reducing children’s pain during dental procedures. Authors compared distraction techniques, indicating no significant differences in effectiveness of active, passive, or combined distraction techniques [98]. Providing examination, observation, and procedural rooms with a simple tool such as soap bubble machines, kaleidoscopes, distraction cards, or more complex ones such as multimedia projectors or audio-players may help to assure a safe and comfortable environment for a child and his or her carers [7]. Canbulat et al. demonstrated that distraction cards and kaleidoscopes significantly reduced pain and anxiety caused by venepuncture in 7–11-year-old children [99]. Music has been shown to have a soothing effect as a therapeutic method. There are different types of music therapy, for example, active or live music, passive music, or music videos which could be used before or during the procedure. All these methods have demonstrated benefits in reducing procedural pain and anxiety [100]. Nguyen et al. showed that music therapy reduced pain and anxiety in children undergoing lumbar puncture [101]. Reduced pain and increased rates of successful venepuncture under the influence of music therapy was proven by Wang et al. [102]. Vosoghi et al. determined benefits of a bubble-maker, demonstrating significant pain relief in an intervention group [103]. Sil et al. noted a significant increase in pain tolerance when children were playing virtual reality videogames during a cold pressor test [97]. A simple conversation about the patient’s friends, leisure, favorite toys, or movies can also make a huge difference in levels of stress and anxiety [102]. To have the greatest effect, distraction techniques should be chosen considering age, development level, and cognitive and communication skills [104].

Distraction techniques for neonates and infants should be passive mostly. It is usually visual or auditory tools such as mirrors, pictures, cartoons, lullabies, or music [105,106]. One of the studies supporting the efficacy of nonpharmacological pain management in neonates was performed by Bo et al. in 2000. The authors determined that non-nutritive sucking used together with music therapy reduced neonatal pain during heel-sticks [105]. Also, a number of studies demonstrated a beneficial effect of oral sucrose on neonatal procedural pain caused by heel-sticks, intramuscular injections, and venepunctures [107,108,109,110]. Further, Acharya et al. published an article showing that oral sucrose not only reduced pain in term but also preterm infants [111]. In addition, breastfeeding during heel lance provides even better analgesia in comparison to oral sucrose, according to a randomized controlled trial performed by Codipietro et al. [112]. The kangaroo method was indicated to have an effect during various procedures in term and preterm neonates as well [113,114]. In a study performed by Gray et al., skin-to-skin contact was proven to reduce pain and decrease arousal in newborns undergoing heel lance [115]. Young children and preschoolers respond best to active techniques, such as blowing bubbles and playing with toys, and passive techniques, such as nonprocedural talking, singing songs, and reading books together with their parents or a medical professional [7]. It is recommended to include school-aged children in making decisions regarding the procedure. Letting them decide if they want to sit or lie down may help them to feel more in control of a stressful situation. Blowing bubbles, singing songs, using relaxation techniques as well as watching videos and television or listening to music may be helpful for school-aged children, too [7,116]. Privacy is extremely important for adolescents, as they can hide or exaggerate their pain in front of others. They should be provided with a choice of their own distraction technique. Nonprocedural conversations, videos, music, and breathing techniques may be beneficial to decrease their pain [7].

According to a variety of neuroimaging studies, distraction techniques activate certain parts of the brain and are associated with a weaker perception of pain. Frankenstein et al. examined healthy volunteers using functional magnetic resonance imaging (fMRI) and determined that distraction during a cold pressor test (application of 0–2 °C compress) resulted in reduced stimulation of the anterior cingulate gyrus subregions responsible for pain experience and increased stimulation of subregions associated with the distraction tasks [117]. Moreover, changes were observed in areas of the brain associated with sensoric and affective motivational perception of pain, including decreased activation of the thalamus, primary and secondary somatosensoric cortices, and anterior cingulate cortex and increased activation in periaquaeductal gray substance, cingulofrontal cortex, and posterior thalamus [117,118,119,120,121].

Different cutaneous stimulation or counter-irritation methods can be used during procedures such as venepuncture or shots. These are hot or cold applications, vibration, or superficial massage. Massage therapy improves circulation in the muscles and more quickly eliminates waste products in the body. Its effect is widely recognised to alleviate pain in different chronic conditions such as rheumatic arthritis, cancer, or fibromyalgia. However, its benefit in acute procedural pain has still not been widely analysed [122]. A significant decrease in pain during the procedure was observed in a group with an additional nonpharmacological treatment method. Aminabadi et al. investigated precooling of the soft tissues before administering local anaesthetic [123]. Discomfort and anxiety were reduced in children who received the precooling application. Another cutaneous stimulation method is vibration therapy. It is a fast-acting, noninvasive option used for mild to moderate pain relief [124]. Cooling-vibration analgesia (CVA) is a combination of cold and high-intensity vibration. This acts as a counter stimulus affecting pain perception during needle-related procedures [125]. CVA activates cold and vibration receptors. It results in activation of inhibitory interneurons in the spinal cord, causing reduction in pain signals which are transmitted via peripheral nociceptic pathways [124]. Baxter et al. demonstrated a beneficial effect of the CVA method used for pain management in 4–18-year-old children undergoing venepuncture [126]. Children were divided into two groups: in the first group, the site of injection was numbed with a vapocoolant spray, whereas the second group received the vapocoolant spray together with the application of a cold vibration device. CVA should not be used over areas of nerve damage or broken skin [127]. Moreover, it is not recommended for such conditions as sensitivity to cold (sickle cell disease or Raynauld’s disease) or if a child is suffering from a cold allergy [125]. Additionally, one study revealed that children who received stroking of the skin close to the injection site before and during injection reported less pain compared to those who did not [128].

## 5. Conclusions and Recommendations

Children of different ages, including neonates, are capable of feeling pain and require analgesia for painful procedures. Effective pain reduction during different procedures in the ER is associated with procedural preplanning, appropriate pain evaluation, parental training, and clear and honest information about the process and associated emotions. Each and every child should be preplanned individually. Gender, personality, temperament, previous painful experiences associated with healthcare settings, family participation and influences, as well as the best-suited nonpharmacological treatment method alone or together with pain medication must be considered. The appropriate medical staff with the necessary skills and experience must participate and ensure the most stress-free, safe, and comfortable environment and process for a child undergoing a medical procedure. It is crucial to choose the correct pain evaluation tool according to the child’s age by assessing the patient’s behaviour and physiological signs. Nonpharmacological treatment methods, such as different distraction tools and techniques or cutaneous stimulation, have shown a beneficial effect during different procedures in the ER. They must be the first option in the preplanning period as well as during and after the procedure. Such methods may reduce the dosage of pain medication or exclude pharmacological pain management at some cases. Methods of nonpharmacological pain management are usually inexpensive, easily accessible, reusable, and may be adapted to any environment.

## Figures and Tables

**Table 1:** The pathways of pain and responses in children.

	Premature Newborn	Adult
**Peripheral nervous system**	Nociceptors are fully developed	Nociceptors are fully developed
	Junctions of nociceptive axons and neurons of spinal cord are disorganized	Junctions of nociceptive axons and neurons of spinal cord are complete and organized
**Spinal cord**	Pathways of a pain signal in spinal cord leading to compromised tactile and nociceptive signal differentiation are disorganized	Innervation has a precise structure, allowing the full differentiation of nociceptive and tactile signals
	Junctions of nociceptive and spinal cord neurons do not function properly, and other sensoric neurons dominate, leading to a pain signal that is not as clear and precise	Junctions of nociceptive and other neurons can fully function. Signal of pain is clear and precise
**Immune system**	Immune reaction stops at predominantly anti-inflammatory phase. It allows development of nociceptive system and the inflammation itself does not occur	Immune system responds with neuroinflammation
**Brain stem**	Descending pain modulatory system starting from nuclei in brain stem is not developed. Ascending excitatory pathways dominate	The response of the spinal cord is balanced by both the inhibitory and excitatory pathways
**Brain**	There is no differentiation of tactile and nociceptive stimuli in brain cortex	Nociceptive and tactile stimuli are well differentiated in brain cortex

**Table 2:** Pain indicators in children. Adapted from https://www.nursingtimes.net/download?ac=3028759 [60].

Behavioural Indicators	Physiological Indicators
Irritability Restlessness Aggressiveness Screaming Crying Sobbing Whimpering Unusual quietness Lethargy Unusual posture Disturbed sleep Loss of appetite Unusual posture Increased clinging	Skin colour/sweating Arterial blood pressure Heart rate Respiratory rate Oxygen saturation Posture Neuroendocrine responses (corticosteroid, growth hormone, cortisol, etc.)

**Table 3:** Age-dependent pain expressions [61].

Age	Comprehension of Pain	Behaviour	Language	Pain Evaluation
6 months	Does not understand pain, responds to stress expressed by parents	Grimaces, generalized movements of limbs and body	Cries	NIPS ^1^/FLACC ^2^
6–12 months	Pain memory already exists, responds to anxiety expressed by parents	Grimaces, irritability, anxiousness, reactions to stimuli are determined by reflexes	Cries	
1–3 years	Does not understand what causes pain and why it occurs	Localized reactions to stimuli, aggression, generalized resistance	Cries, screams. Cannot describe intensity or type of pain	FLACC ^2^
3–6 years	Understands pain but does not connect it with a disease (may connect it with trauma). Does not understand how a painful procedure can help them	Active physical resistance, aggressive behaviour, verbal and physical response to pain.	Has the ability to describe pain, its localization, intensity. Sometime denies pain	FLACC ^2^/FPS-R ^3^
7–9 years	Does not understand reasons of pain but can connect pain with disease. May understand the benefits of painful procedures	Bargaining, passive resistance, tense body, emotional withdrawal	Can localize the pain precisely, has the skills to describe its intensity, type, and connections with body parts	FPS-R ^3^/VAS ^4^/CAS ^5^
10–12 years	Has a better understanding of disease’s/trauma’s relations to pain	Sometimes pretends to feel well in order to demonstrate courage	Can describe the localization and intensity of pain well	
13–18 years	Complex understanding of pain and its reasons. Ability to recognize qualitative and quantitative characteristics of pain	Tries to act like adults, may not complain because of clues of medical staff	The older a child, the more complex their pain descriptions are. May think that everybody knows and understands their pain so there is no need to talk about it	

^1^—NIPS—Neonatal Infant Pain Scale; ^2^—FLACC—Face, Legs, Activity, Cry, and Consolability scale; ^3^—FPS-R—Faces Pain Scale Revised; ^4^—VAS—Visual Analogue Scale; ^5^—CAS—Color Analog Scale.

**Table 4:** Pain evaluation for babies and newborns (NIPS scale).

	0 Points	1 Point	2 Points
Facial expression	Relaxed	Contracted	
Cry	Absent	Mumbling	Vigorous
Breathing	Relaxed	Different than basal	
Arms	Relaxed	Flexed/stretched	
Legs	Relaxed	Flexed/stretched	
Alertness	Sleeping/calm	Uncomfortable	

**Table 5:** Face, Legs, Activity, Cry, and Consolability (FLACC) scale.

	Scoring		
	0	1	2
Face	No particular expression/smiles/disinterested	Withdrawn, shows occasional grimace, or frown	Frequent or constant frown, clenched jaw, quivering chin
Legs	Normal position/relaxed	Restless, tense, uneasy	Kicking/drawn up
Activity	Normal position/moves easily/lying quietly	Squirming, shifting back and forth, tense	Arched/rigid/jerking
Cry	Does not cry	Moans or whimpers, occasional complaint	Cries steadily, screams or sobs, frequent complaints
Consolability	Relaxed/content	Calmed by occasional touching, hugging, being talked to. Distractible	Difficult to console or comfort

**Table 6:** Examples of verbal communication with children undergoing a painful procedure.

Language to Avoid	Recommended Language
“Don’t cry”/“Don’t act like a baby”/“There is nothing to be scared of”	Encouraging: “You are so brave”/“I am proud of you”/“Well done”
“If you don’t listen I will draw your blood”/“The nurse is hurting you so bad, poor baby”	Explaining: “The medication will work better if we will let them into your vein”/“It will help you to feel better”
“Everything will be done soon”	Being clear and specific: “It will take as long as your favourite cartoon”/“It will be shorter than a ride home”
“It will be painful”/“You will not feel anything”	Telling the truth: “You might feel a slight pinch”
“Everything will be okay”/“Tell me when you’re ready”/“I am sorry”	Distracting: “What is your favourite movie?”/“What is the name of your best friend?”
“I will clean your hand with antiseptic.”	Procedural and sensory information: “You may feel a cold and wet pad while I clean your hand with antiseptic.”

**Table 7:** Examples of passive and active distraction techniques.

Passive Distraction (The Patient does not Participate in the Process of Distraction)	Active Distraction (The Patient Participates Pctively)
Mirrors Pictures Lullabies Music Kangaroo method Cartoons Colourful walls Procedural talking	Kaleidoscope Virtual reality Cards Bubble machines Toys Videogames Coloring books, etc.

## References

[1] IASPTerminology-IASP. http://www.iasppain.org/Education/Content.aspx?ItemNumber=1698&navItemNumber=576#Pain.

[2] Wier L.M., Yu H., Owens P.L., Washington R. (2013). Overview of Children in the Emergency Department, 2010: Statistical Brief #157. https://www.ncbi.nlm.nih.gov/pubmed/24006551.

[3] Ortiz M.I., López-Zarco M., Arreola-Bautista E.J. (2012). Procedural Pain and Anxiety in Paediatric Patients in a Mexican Emergency Department. J. Adv. Nurs..

[4] MacLean S., Obispo J., Young K.D. (2007). The Gap between Pediatric Emergency Department Procedural Pain Management Treatments Available and Actual Practice. Pediatr. Emerg. Care.

[5] Ali S., Chambers A., Johnson D., Newton A., Vandermeer B., Williamson J., Curtis S.J. (2014). Reported practice variation in pediatric pain management: A survey of Canadian pediatric emergency physicians. CJEM.

[6] Stevens B.J., Abbott L.K., Yamada J., Harrison D., Stinson J., Taddio A., Barwick M., Latimer M., Scott S.D., Rashotte J. (2011). Epidemiology and Management of Painful Procedures in Children in Canadian Hospitals. CMAJ.

[7] Srouji R., Ratnapalan S., Schneeweiss S. (2010). Pain in Children: Assessment and Nonpharmacological Management. Int. J. Pediatr..

[8] Walco G.A., Cassidy R.C., Schechter N.L. (1994). Pain, Hurt, and Harm—The Ethics of Pain Control in Infants and Children. N. Engl. J. Med..

[9] Schechter N.L. (1989). The Undertreatment of Pain in Children: An Overview. Pediatr. Clin. N. Am..

[10] Edmonds J., Twycross A. (2018). Mothers’ Experiences of Managing Their Child’s Pain before and during Attendance at the Emergency Department. J. Clin. Nurs..

[11] Smith R.W., Shah V., Goldman R.D., Taddio A. (2007). Caregivers’ Responses to Pain in Their Children in the Emergency Department. Arch. Pediatr. Adolesc. Med..

[12] Dahlquist L.M., Busby S.M., Slifer K.J., Tucker C.L., Eischen S., Hilley L., Sulc W. (2002). Distraction for Children of Different Ages Who Undergo Repeated Needle Sticks. J. Pediatr. Oncol. Nurs..

[13] Bergomi P., Scudeller L., Pintaldi S., Dal Molin A. (2018). Efficacy of Non-Pharmacological Methods of Pain Management in Children Undergoing Venipuncture in a Pediatric Outpatient Clinic: A Randomized Controlled Trial of Audiovisual Distraction and External Cold and Vibration. J. Pediatr. Nurs..

[14] Fitzgerald M. (2005). The Development of Nociceptive Circuits. Nat. Rev. Neurosci..

[15] Kėvelaitis E., Illert M., Hultborn H., Miliauskas R., Abraitis R., Cibas P., Gutmanas A., Skurvydas A., Stasiulis A., Lažauskas R. (2006). Žmogaus Fiziologija.

[16] Anand K., Stevens B., McGrath P.J. (2007). Pain in Neonates and Infants: Pain Research and Clinical Management Series.

[17] Bartocci M., Bergqvist L.L., Lagercrantz H., Anand K.J. (2006). Pain activates cortical areas in the preterm newborn brain. Pain.

[18] Slater R., Fabrizi L., Worley A., Meek J., Boyd S., Fitzgerald M. (2010). Premature infants display increased noxious-evoked neuronal activity in the brain compared to healthy age-matched term-born infants. Neuroimage.

[19] Fabrizi L., Slater R., Worley A., Meek J., Boyd S., Olhede S., Fitzgerald M. (2011). A shift in sensory processing that enables the developing human brain to discriminate touch from pain. Curr. Biol..

[20] Holsti L., Grunau R.E., Oberlander T.F., Whitfield M.F. (2005). Prior pain induces heightened motor responses during clustered care in preterm infants in the NICU. Early Hum. Dev..

[21] Boissé L., Spencer S., Mouihate A., Vergnolle N., Pittman Q. (2005). Neonatal immune challenge alters nociception in the adult rat. Pain.

[22] Beggs S., Currie G., Salter M.W., Fitzgerald M., Walker S.M. (2012). Priming of Adult Pain Responses by Neonatal Pain Experience: Maintenance by Central Neuroimmune Activity. Brain.

[23] Schwaller F., Fitzgerald M. (2014). The consequences of pain in early life: Injury-induced plasticity in developing pain pathways. Eur. J. Neurosci..

[24] Brummelte S., Grunau R.E., Chau V., Poskitt K.J., Brant R., Vinall J., Gover A., Synnes A.R., Miller S.P. (2012). Procedural pain and brain development in premature newborns. Ann. Neurol..

[25] Fitzgerald M., Hanson M. (1991). The development of descending brainstem control of spinal cord sensory processing. Foetal and Neonatal Brainstem: Development and Clinical Issues.

[26] Fitzgerald M., Koltzenburg M. (1986). The Functional Development of Descending Inhibitory Pathways in the Dorsolateral Funiculus of the Newborn Rat Spinal Cord. Dev. Brain Res..

[27] Schulte F., Linneweh F. (1968). Gestation, wachsturn und hirnentwicklung. Fortscritte der Paedologie.

[28] Lundeberg S., Lundeberg T. (2013). Pain in Infants and Children—Physiological Background and Clinical Aspects. Acupunct. Relat. Ther..

[29] Bouza H. (2009). The impact of pain in the immature brain. J. Matern.-Fetal Neonatal Med..

[30] Smith G.C., Gutovich J., Smyser C., Pineda R., Newnham C., Tjoeng T.H., Vavasseur C., Wallendorf M., Neil J., Inder T. (2011). Neonatal intensive care unit stress is associated with brain development in preterm infants. Ann. Neurol..

[31] Tombini M., Pasqualetti P., Rizzo C., Zappasodi F., Dinatale A., Seminara M., Ercolani M., Rossini P.M., Agostino R. (2009). Extrauterine maturation of somatosensory pathways in preterm infants: A somatosensory evoked potential study. Clin. Neurophysiol..

[32] Vanhatalo S., Jousmäki V., Andersson S., Metsäranta M. (2009). An easy and practical method for routine, bedside testing of somatosensory systems in extremely low birth weight infants. Pediatr. Res..

[33] Lupien S.J., McEwen B.S., Gunnar M.R., Heim C. (2009). Effects of stress throughout the lifespan on the brain, behaviour and cognition. Nat. Rev. Neurosci..

[34] Anand K.J., Coskun V., Thrivikraman K.V., Nemeroff C.B., Plotsky P.M. (1999). Long-term behavioral effects of repetitive pain in neonatal rat pups. Physiol. Behav..

[35] Dührsen L., Simons S.H., Dzietko M., Genz K., Bendix I., Boos V., Sifringer M., Tibboel D., Felderhoff-Mueser U. (2013). Effects of repetitive exposure to pain and morphine treatment on the neonatal rat brain. Neonatology.

[36] Van den Hoogen N., Patijn J., Tibboel D., Joosten B., Fitzgerald M., Kwok C. (2018). Repeated touch and needle-prick stimulation in the neonatal period increases the baseline mechanical sensitivity and postinjury hypersensitivity of adult spinal sensory neurons. Pain.

[37] Van den Hoogen N., Tibboel D., Honig W., Hermes D., Patijn J., Joosten E. (2016). Neonatal paracetamol treatment reduces long-term nociceptive behaviour after neonatal procedural pain in rats. Eur. J. Pain.

[38] Koch S., Fitzgerald M. (2013). Activity-dependent development of tactile and nociceptive spinal cord circuits. Ann. N. Y. Acad. Sci..

[39] LaPrairie J. (2009). Neonatal injury alters adult pain sensitivity by increasing opioid tone in the periaqueductal gray. Front. Behav. Neurosci..

[40] Chen M., Xia D., Min C., Zhao X., Chen Y., Liu L., Xiaonan L. (2016). Neonatal repetitive pain in rats leads to impaired spatial learning and dysregulated hypothalamic-pituitary-adrenal axis function in later life. Sci. Rep..

[41] Zwicker J.G., Grunau R.E., Adams E., Chau V., Brant R., Poskitt K.J., Synnes A., Miller S.P. (2013). Score for neonatal acute physiology-II and neonatal pain predict corticospinal tract development in premature newborns. Pediatr. Neurol..

[42] Doesburg S.M., Chau C.M., Cheung T.P., Moiseev A., Ribary U., Herdman A.T., Miller S.P., Cepeda I.L., Synnes A., Grunau R.E. (2013). Neonatal pain-related stress, functional cortical activity and visual-perceptual abilities in school-age children born at extremely low gestational age. Pain.

[43] Vinall J., Grunau R., Bjornson B. Impact of neonatal pain-related stress on brain and IQ at school age in children born preterm. Pediatrics, Poster presentation. Proceedings of the 9th International Forum on Pediatric Pain.

[44] Ranger M., Chau C.M., Garg A., Woodward T.S., Beg M.F., Bjornson B., Poskitt K., Fitzpatrick K., Synnes A.R., Miller S.P. (2013). Neonatal pain-related stress predicts cortical thickness at age 7 years in children born very preterm. PLoS ONE.

[45] Hohmeister J., Kroll A., Wollgarten-Hadamek I., Zohsel K., Demirakça S., Flor H., Hermann C. (2010). Cerebral processing of pain in school-aged children with neonatal nociceptive input: An exploratory fMRI study. Pain.

[46] Chrousos G. (2009). Stress and disorders of the stress system. Nat. Rev. Endocrinol..

[47] Soriano S.G., Anand K.J.S. (2005). Anesthetics and Brain Toxicity. Curr. Opin. Anaesthesiol..

[48] Atici S., Cinel L., Cinel I., Doruk N., Aktekin M., Akca A., Camdeviren H., Oral U. (2004). Opioid neurotoxicity: Comparison of morphine and tramadol in an experimental rat model. Int. J. Neurosci..

[49] Steinhorn R., McPherson C., Anderson P., Neil J., Doyle L., Inder T. (2015). Neonatal Morphine Exposure in Very Preterm Infants—Cerebral Development and Outcomes. J. Pediatr..

[50] Ferguson S.A., Ward W.L., Paule M.G., Hall R.W., Anand K.J. (2012). A pilot study of preemptive morphine analgesia in preterm neonates: Effects on head circumference, social behavior, and response latencies in early childhood. Neurotoxicol. Teratol..

[51] Jin J., Gong K., Zou X., Wang R., Lin Q., Chen J. (2013). The blockade of NMDA receptor ion channels by ketamine is enhanced in developing rat cortical neurons. Neurosci. Lett..

[52] Vutskits L., Xie Z. (2016). Lasting impact of general anaesthesia on the brain: Mechanisms and relevance. Nat. Rev. Neurosci..

[53] Dong C., Anand K. (2013). Developmental neurotoxicity of ketamine in pediatric clinical use. Toxicol. Lett..

[54] Stinson J.N., Kavanagh T., Yamada J., Gill N., Stevens B. (2006). Systematic Review of the Psychometric Properties, Interpretability and Feasibility of Self-Report Pain Intensity Measures for Use in Clinical Trials in Children and Adolescents. Pain.

[55] Blount R.L., Loiselle K.A. (2009). Behavioural Assessment of Pediatric Pain. Pain Res. Manag..

[56] Nilsson S., Finnström B., Kokinsky E. (2008). The FLACC Behavioral Scale for Procedural Pain Assessment in Children Aged 5–16 Years. Paediatr. Anaesth..

[57] Hunt A., Wisbeach A., Seers K., Goldman A., Crichton N., Perry L., Mastroyannopoulou K. (2007). Development of the Paediatric Pain Profile: Role of Video Analysis and Saliva Cortisol in Validating a Tool to Assess Pain in Children with Severe Neurological Disability. J. Pain Symptom Manag..

[58] Buttner W., Finke W., Büttner W., Finke W. (2000). Analysis of Behavioural and Physiological Parameters for the Assessment of Postoperative Analgesic Demand in Newborns, Infants and Young Children: A Comprehensive Report on Seven Consecutive Studies. Paediatr. Anaesth..

[59] Brudvik C., Moutte S.-D., Baste V., Morken T. (2017). A Comparison of Pain Assessment by Physicians, Parents and Children in an Outpatient Setting. Emerg. Med. J..

[60] Nursing Times. https://www.nursingtimes.net/download?ac=3028759.

[61] McGrath P. (1995). Pain in the Pediatric Patient: Practical Aspects of Assessment. Pediatr. Ann..

[62] Lawrence J., Alcock D., McGrath P., Kay J., MacMurray S.B., Dulberg C. (1993). The Development of a Tool to Assess Neonatal Pain. Neonatal Netw..

[63] Merkel S.I., Voepel-Lewis T., Shayevitz J.R., Malviya S. (1997). The FLACC: A Behavioral Scale for Scoring Postoperative Pain in Young Children. Pediatr. Nurs..

[64] Voepel-Lewis T., Zanotti J., Dammeyer J.A., Merkel S. (2010). Reliability and Validity of the Face, Legs, Activity, Cry, Consolability Behavioral Tool in Assessing Acute Pain in Critically Ill Patients. Am. J. Crit. Care.

[65] Hicks C.L., Von Baeyer C.L., Spafford P.A., Van Korlaar I., Goodenough B. (2001). The Faces Pain Scale—Revised: Toward a Common Metric in Pediatric Pain Measurement. Pain.

[66] Bieri D., Reeve R.A., Champion G.D., Addicoat L., Zegler J.B. (1990). The Faces Pain Scale for the self-assessment of the severity of pain experienced by children: Development, initial validation, and preliminary investigation for ratio scale properties. Pain.

[67] Price D.D., McGrath P.A., Rafii A., Buckingham B. (1983). The Validation of Visual Analogue Scales as Ratio Scale Measures for Chronic and Experimental Pain. Pain.

[68] Tsze D.S., von Baeyer C.L., Bulloch B., Dayan P.S. (2013). Validation of Self-Report Pain Scales in Children. Pediatrics.

[69] Hawker G.A., Mian S., Kendzerska T., French M. (2011). Measures of Adult Pain: Visual Analog Scale for Pain (VAS Pain), Numeric Rating Scale for Pain (NRS Pain), McGill Pain Questionnaire (MPQ), Short-Form McGill Pain Questionnaire (SF-MPQ), Chronic Pain Grade Scale (CPGS), Short Form-36 Bodily Pain Scale (SF-36 BPS), and Measure of Intermittent and Constant Osteoarthritis Pain (ICOAP). Arthritis Care Res..

[70] McGrath P.A., Seifert C.E., Speechley K.N., Booth J.C., Stitt L., Gibson M.C. (1996). A New Analogue Scale for Assessing Children’s Pain: An Initial Validation Study. Pain.

[71] Le May S., Ballard A., Khadra C., Gouin S., Plint A.C., Villeneuve E., Mâsse B., Tsze D.S., Neto G., Drendel A.L. (2018). A Comparison of the Psychometric Properties of Three Pain Scales Used in the Pediatric Emergency Department. Pain.

[72] Fernando C., Rifaya M., Asantha W., Chandrarathna R., Wijeratna A. (2017). A comparison of three self-report pain scales in Sri Lankan children. Sri Lanka J. Child Health..

[73] Goodenough B., Piira T., von Baeyer C., Chua K., Wu E., Trieu J., Champion G.D. (2005). Comparing six self-report measures of pain intensity in children. Suff. Child..

[74] Spafford P.A., Von Baeyer C.L., Hicks C.L. (2002). Expected and Reported Pain in Children Undergoing Ear Piercing: A Randomized Trial of Preparation by Parents. Behav. Res. Ther..

[75] Kolk A.M.M., Van Hoof R., Fiedeldij Dop M.J.C. (2000). Preparing Children for Venepuncture. The Effect of an Integrated Intervention on Distress before and during Venepuncture. Child Care Health Dev..

[76] Suls J., Wan C.K. (1989). Effects of Sensory and Procedural Information on Coping with Stressful Medical Procedures and Pain: A Meta-Analysis. J. Consult. Clin. Psychol..

[77] Smith L., Callery P. (2005). Children’s Accounts of Their Preoperative Information Needs. J. Clin. Nurs..

[78] Crittenden P.M.K., Dallos R. (2009). All in the Family: Integrating Attachment and Family Systems Theories. Clin. Child Psychol. Psychiatry.

[79] Walsh B.M., Bartfield J.M. (2006). Survey of Parental Willingness to Pay and Willingness to Stay for “Painless” Intravenous Catheter Placement. Pediatr. Emerg. Care.

[80] Mangurten J., Scott S.H., Guzzetta C.E., Clark A.P., Vinson L., Sperry J., Hicks B., Voelmeck W. (2006). Effects of Family Presence During Resuscitation and Invasive Procedures in a Pediatric Emergency Department. J. Emerg. Nurs..

[81] Blount R.L., Bachanas P.J., Powers S.W., Cotter M.C., Franklin A., Chaplin W., Mayfield J., Henderson M., Blount S.D. (1992). Training Children to Cope and Parents to Coach Themduring Routine Immunizations: Effects on Child, Parent, and Staff Behaviors. Behav. Ther..

[82] Pedro H., Barros L., Pereira A.I. (2016). Pediatric Immunization Distress: A Cluster Analyses of Children’s, Parents’, and Nurses’ Behaviors during the Anticipatory Phase. Clin. J. Pain.

[83] Bush J.P., Cockrell C.S. (1987). Maternal Factors Predicting Parenting Behaviors in the Pediatric Clinic. J. Pediatr. Psychol..

[84] Bearden D.J., Feinstein A., Cohen L.L. (2012). The Influence of Parent Preprocedural Anxiety on Child Procedural Pain: Mediation by Child Procedural Anxiety. J. Pediatr. Psychol..

[85] Frank N.C., Blount R.L., Smith A.J., Manimala M.R., Martin J.K. (1995). Society of Pediatric Psychology Student Research Award: Parent and Staff Behavior, Previous Child Medical Experience, and Maternal Anxiety as They Relate to Child Procedural Distress and Coping. J. Pediatr. Psychol..

[86] Robinson P.S., Green J. (2015). Ambient versus Traditional Environment in Pediatric Emergency Department. Heal. Environ. Res. Des. J..

[87] Monti F., Agostini F., Dellabartola S., Neri E., Bozicevic L., Pocecco M. (2012). Pictorial Intervention in a Pediatric Hospital Environment: Effects on Parental Affective Perception of the Unit. J. Environ. Psychol..

[88] Stephens B.K., Barkey M.E., Hall H.R. (1999). Techniques to Comfort Children during Stressful Procedures. Accid. Emerg. Nurs..

[89] Howard R., Carter R., Curry J., Jain A., Liossi C., Morton N., Rivett K., Rose M., Tyrrell J., Walker S. (2012). Good Practice in Postoperative and Procedural Pain Management. Pediatr. Anesth..

[90] Cohen L.L., MacLaren J.E., Fortson B.L., Friedman A., DeMore M., Lim C.S., Shelton E., Gangaram B. (2006). Randomized Clinical Trial of Distraction for Infant Immunization Pain. Pain.

[91] Cohen L.L. (2008). Behavioral Approaches to Anxiety and Pain Management for Pediatric Venous Access. Pediatrics.

[92] Foster M.E. (2011). Intranasal Fentanyl and Midazolam Use in a Pediatric Emergency Department. Pharmacotherapy.

[93] Duerden E.G., Guo T., Dodbiba L., Chakravarty M.M., Chau V., Poskitt K.J., Synnes A., Grunau R.E., Miller S.P. (2016). Midazolam Dose Correlates with Abnormal Hippocampal Growth and Neurodevelopmental Outcome in Preterm Infants. Ann. Neurol..

[94] Legrain V., Van Damme S., Eccleston C., Davis K.D., Seminowicz D.A., Crombez G.A. (2009). Neurocognitive Model of Attention to Pain: Behavioral and Neuroimaging Evidence. Pain.

[95] Eccleston C., Crombez G. (1999). Pain Demands Attention: A Cognitive-Affective Model of the Interruptive Function of Pain. Psychol. Bull..

[96] Law E.F., Dahlquist L.M., Sil S., Weiss K.E., Herbert L.J., Wohlheiter K., Horn S.B. (2011). Videogame Distraction Using Virtual Reality Technology for Children Experiencing Cold Pressor Pain: The Role of Cognitive Processing. J. Pediatr. Psychol..

[97] Sil S., Dahlquist L.M., Thompson C., Hahn A., Herbert L., Wohlheiter K., Horn S. (2014). The Effects of Coping Style on Virtual Reality Enhanced Videogame Distraction in Children Undergoing Cold Pressor Pain. J. Behav. Med..

[98] Abdelmoniem S.A., Mahmoud S.A. (2016). Comparative Evaluation of Passive, Active, and Passive-Active Distraction Techniques on Pain Perception during Local Anesthesia Administration in Children. J. Adv. Res..

[99] Canbulat N., Inal S., Sönmezer H. (2014). Efficacy of Distraction Methods on Procedural Pain and Anxiety by Applying Distraction Cards and Kaleidoscope in Children. Asian Nurs. Res. (Korean Soc. Nurs. Sci.).

[100] Klassen J.A., Liang Y., Tjosvold L., Klassen T.P., Hartling L. (2008). Music for Pain and Anxiety in Children Undergoing Medical Procedures: A Systematic Review of Randomized Controlled Trials. Ambul. Pediatr..

[101] Nguyen T.N., Nilsson S., Hellström A.-L., Bengtson A. (2010). Music Therapy to Reduce Pain and Anxiety in Children with Cancer Undergoing Lumbar Puncture: A Randomized Clinical Trial. J. Pediatr. Oncol. Nurs..

[102] Wang Z.X., Sun L.H., Chen A.P. (2008). The Efficacy of Non-Pharmacological Methods of Pain Management in School Age Children Receiving Venepuncture in a Paediatric Department: A Randomized Controlled Trial of Audiovisual Distraction and Routine Psychological Intervention. Swiss Med. Wkly..

[103] Vosoghi N., Chehrzad M., Abotalebi G., Roshan Z.A. (2010). Effects of Distraction on Physiologic Indices and Pain Intensity in Children Aged 3–6 Undergoing IV Injection. Hayat.

[104] Vessey J.A., Carlson K.L., McGill J. (1994). Use of Distraction with Children during an Acute Pain Experience. Nurs. Res..

[105] Bo L.K., Callaghan P. (2000). Soothing Pain-Elicited Distress in Chinese Neonates. Pediatrics.

[106] Cohen L.L. (2002). Reducing Infant Immunization Distress through Distraction. Heal. Psychol..

[107] Basnet S., Shrestha L., Shrestha P.S. (2010). Sucrose as an Analgesic in Relieving Procedural Pain in Neonates. J. Neonatal-Perinat. Med..

[108] Storm H., Fremming A. (2002). Food Intake and Oral Sucrose in Preterms Prior to Heel Prick. Acta Paediatr. Int. J. Paediatr..

[109] Taddio A., Shah V., Hancock R., Smith R.W., Stephens D., Atenafu E., Beyene J., Koren G., Stevens B., Katz J. (2008). Effectiveness of Sucrose Analgesia in Newborns Undergoing Painful Medical Procedures. CMAJ.

[110] Haouari N., Wood C., Griffiths G., Levene M. (1995). The Analgesic Effect of Sucrose in Full Term Infants: A Randomised Controlled Trial. BMJ.

[111] Acharya A.B. (2004). Oral Sucrose Analgesia for Preterm Infant Venepuncture. Arch. Dis. Child.-Fetal Neonatal Ed..

[112] Codipietro L., Ceccarelli M., Ponzone A. (2008). Breastfeeding or Oral Sucrose Solution in Term Neonates Receiving Heel Lance: A Randomized, Controlled Trial. Pediatrics.

[113] Johnston C.C., Stevens B., Pinelli J., Gibbins S., Filion F., Jack A., Steele S., Boyer K., Veilleux A. (2003). Kangaroo Care Is Effective in Diminishing Pain Response in Preterm Neonates. Arch. Pediatr. Adolesc. Med..

[114] Kostandy R.R., Anderson G.C., Good M. (2013). Skin-to-Skin contact diminishes pain from hepatitis B vaccine injection in healthy full-term neonates. Neonatal Netw..

[115] Gray L., Watt L., Blass E.M. (2000). Skin-to-Skin Contact Is Analgesic in Healthy Newborns. Pediatrics.

[116] Bellieni C.V., Cordelli D.M., Raffaelli M., Ricci B., Morgese G., Buonocore G. (2006). Analgesic Effect of Watching TV during Venipuncture. Arch. Dis. Child..

[117] Frankenstein U.N., Richter W., McIntyre M.C., Rémy F. (2001). Distraction Modulates Anterior Cingulate Gyrus Activations during the Cold Pressor Test. Neuroimage.

[118] Hoffman H.G., Richards T.L., Coda B., Bills A.R., Blough D., Richards A.L., Sharar S.R. (2004). Modulation of Thermal Pain-Related Brain Activity with Virtual Reality: Evidence from FMRI. Neuroreport.

[119] Jackson T., Yang Z., Li X., Chen H., Huang X., Meng J. (2012). Coping When Pain Is a Potential Threat: The Efficacy of Acceptance versus Cognitive Distraction. Eur. J. Pain.

[120] Petrovic P., Petersson K.M., Ghatan P.H., Stone-Elander S., Ingvar M. (2000). Pain-Related Cerebral Activation Is Altered by a Distracting Cognitive Task. Pain.

[121] Valet M., Sprenger T., Boecker H., Willoch F., Rummeny E., Conrad B., Erhard P., Tolle T.R. (2004). Distraction Modulates Connectivity of the Cingulo-Frontal Cortex and the Midbrain during Pain—An FMRI Analysis. Pain.

[122] Hernandez-Reif M., Field T., Largie S., Hart S., Redzepi M., Nierenberg B., Peck M. (2001). Childrens’ Distress during Burn Treatment Is Reduced by Massage Therapy. J. Burn Care Rehabil..

[123] Aminabadi N.A., Farahani R.M.Z. (2009). The Effect of Pre-Cooling the Injection Site on Pediatric Pain Perception during the Administration of Local Anesthesia. J. Contemp. Dent. Pract..

[124] Baba L.R., McGrath J.M., Liu J. (2010). The Efficacy of Mechanical Vibration Analgesia for Relief of Heel Stick Pain in Neonates: A Novel Approach. J. Perinat. Neonatal Nurs..

[125] Schreiber S., Cozzi G., Rutigliano R., Assandro P., Tubaro M., Cortellazzo Wiel L., Ronfani L., Barbi E. (2016). Analgesia by Cooling Vibration during Venipuncture in Children with Cognitive Impairment. Acta Paediatr. Int. J. Paediatr..

[126] Baxter A.L., Cohen L.L., McElvery H.L., Lawson M.L., von Baeyer C.L. (2011). An Integration of Vibration and Cold Relieves Venipuncture Pain in a Pediatric Emergency Department. Pediatr. Emerg. Care.

[127] Inal S., Kelleci M. (2012). Relief of Pain during Blood Specimen Collection in Pediatric Patients. MCN Am. J. Matern. Nurs..

[128] Sparks L.G. (1998). A Comparison of the Effects of Cutaneous Stimulation and Distraction on Children’s Perceptions of Injection Pain. Ph.D. Thesis.

